# Recent Progress in the Synthesis of MoS_2_ Thin Films for Sensing, Photovoltaic and Plasmonic Applications: A Review

**DOI:** 10.3390/ma14123283

**Published:** 2021-06-14

**Authors:** Driss Mouloua, Ahmed Kotbi, Geetanjali Deokar, Khaled Kaja, Mimoun El Marssi, My Ali EL Khakani, Mustapha Jouiad

**Affiliations:** 1 Laboratory of Physics of Condensed Matter, University of Picardie Jules Verne, 33 Saint Leu, 80039 Amiens, France; driss.mouloua@etud.u-picardie.fr (D.M.); ahmed.kotbi@u-picardie.fr (A.K.); mimoun.elmarssi@u-picardie.fr (M.E.M.); 2 Institut National de la Recherche Scientifique, Centre-Énergie, Matériaux et Télécommunications, 1650, Blvd, Lionel–Boulet, Varennes, QC J3X-1S2, Canada; 3 Physical Science and Engineering Division, Kaust University, Thuwal 23955-6900, Saudi Arabia; geetanjali.deokar@kaust.edu.sa; 4 Laboratoire National de métrologie et d’essais (LNE), 29 av. Roger Hannequin, 78197 Trappes, France; khaled.kaja@lne.fr

**Keywords:** layered materials, 2D-MoS_2_, pulsed laser deposition, chemical vapor deposition, photovoltaic, gas sensors, plasmonics

## Abstract

In the surge of recent successes of 2D materials following the rise of graphene, molybdenum disulfide (2D-MoS_2_) has been attracting growing attention from both fundamental and applications viewpoints, owing to the combination of its unique nanoscale properties. For instance, the bandgap of 2D-MoS_2_, which changes from direct (in the bulk form) to indirect for ultrathin films (few layers), offers new prospects for various applications in optoelectronics. In this review, we present the latest scientific advances in the field of synthesis and characterization of 2D-MoS_2_ films while highlighting some of their applications in energy harvesting, gas sensing, and plasmonic devices. A survey of the physical and chemical processing routes of 2D-MoS_2_ is presented first, followed by a detailed description and listing of the most relevant characterization techniques used to study the MoS_2_ nanomaterial as well as theoretical simulations of its interesting optical properties. Finally, the challenges related to the synthesis of high quality and fairly controllable MoS_2_ thin films are discussed along with their integration into novel functional devices.

## 1. Introduction

**Figure 1:**
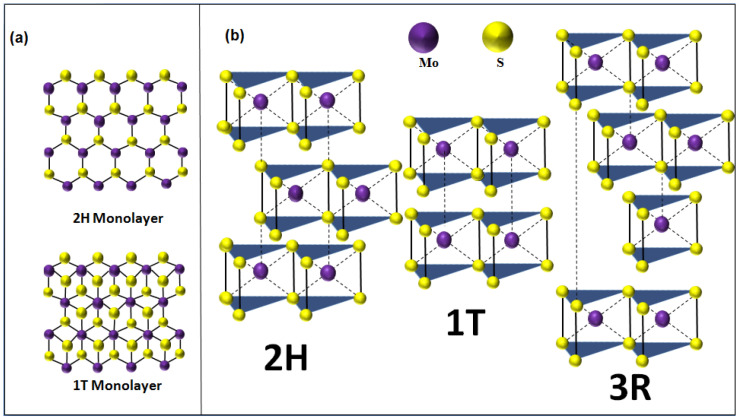
(**a**) Top view of 2H/1T MoS_2_ monolayer. (**b**) Polymorphic structures of MoS_2_ (2H is the hexagonal crystal form, 1T is the tetragonal crystal form, and 3R is the rhombohedral crystal form).

Two-dimensional (2D) materials are generally defined as crystalline substances with a few atoms thickness [1]. Graphene was the first 2D crystal to be ever isolated in 2004 and has since been extensively investigated by many groups around the world [2,3,4,5,6]. In fact, graphene became known as the material of superlatives showing a mechanical strength hundreds of times larger than steel [7] while maintaining a high mechanical flexibility [8] and superior electrical and thermal conductivities [9]. Following the discovery of grapheme [10], a very large spectrum of 2D materials possessing a wide range of highly attractive properties have emerged [8,10]. For instance, two-dimensional transition metal dichalcogenide (2D-TMDs) semiconducting (SC) materials have exhibited unique optical and electrical properties [11,12], resulting from the quantum confinement effect attributed to their shapes and sizes with respect to the Bohr radius [13,14,15,16,17], in addition to their surface effects, which is due to the transition from an indirect bandgap in the “bulk form” to a direct bandgap for the “mono- to few-layer” ultrathin film form [18]. The layered configuration of the 2D-TMDs materials is at the origin of their strong interaction with light [19] and the relatively high mobility of their charge carriers [20], which in turn prompted their use in many optoelectronic applications, such as ultra-thin field-effect transistors [21], photo-detectors [22], light emitting diode [23], and solar-cells [24]. Generally, 2D-TMDs form a family of graphite-like layered thin semiconducting structures with the chemical formula of MX_2_, where M refers to a transition metal atom (Mo, W, etc.) and X is a chalcogen atom (Se, S, etc.). The layered nature of this class of 2D materials induces a strong anisotropy in their electrical, chemical, mechanical, and thermal properties. In particular, molybdenum disulfide (MoS_2_) is the most studied layered 2D-TMD [25,26,27,28,29,30]. From a crystalline point of view, layered MoS_2_ exists in three polymorphic crystalline structures: 1T (tetragonal) [31], 2H (hexagonal) [32], and 3R (rhombohedral) [33] (Figure 1). The crystallographic parameters associated to these crystalline forms are summarized in Table 1. In the case of mono- to few-layer structures, 2H-MoS_2_ is the most thermodynamically stable phase and thus the most commonly encountered. When the MoS_2_ is in the monolayer form, it takes an octahedral or a trigonal prismatic coordination phase.

Furthermore, MoS_2_ layered materials were observed to exhibit various shapes and morphologies, such as planar [34,35,36] and vertically aligned nanosheets (NSs) [37], nanoflowers [38], nanotubes [39], nanowires [40], and nanoplatelets [41,42]. This variety of forms could be controlled by choosing suitable synthesis routes with optimized operating parameters [38,39,40,41,43,44,45,46,47]. Thus, it is possible to adjust the 2D-MoS_2_ properties to develop high performance devices i energy storage [47], electronics [46], photonics [45], sensing [48], and field emission [49] applications. Recently, up to few-layer MoS_2_ nanosheets have been shown to be highly efficient for electronic, optoelectronic, and solar energy harvesting devices [50,51,52] because of their tunable direct bandgap [53], strong light-absorption, and prominent photoluminescence with energies lying in the visible range (1.8–1.9 eV) [54].

Although Mo and S are strongly covalently bonded within an individual layer, adjacent sheets are linked together only by the very weak van der Waals interaction. This weak bonding provides a facile processing route such as mechanical or chemical exfoliation to form few- to monolayer MoS_2_ films. Unlike graphene, 2D-MoS_2_ is much less prone to surface contaminations, which offers a superior chemical stability to 2D-MoS2, making it more attractive for the above-mentioned applications [55,56,57].

This review is timely to report on the state of the art of 2D-MoS_2_ from synthesis, properties, and applications viewpoints. It also intends to provide insights on the remaining challenges to widen the applications range of this fantastic 2D-MoS_2_ material. It is organized as follows. In Section 2, various fabrication routes are highlighted with a special focus on physical vapor deposition (PVD) methods. Key processing parameters are pinpointed and their influence on the material characteristics, i.e., thickness, crystallinity, morphology, etc., and properties are underlined. In Section 3, relevant techniques used to investigate the complex structure and morphology of 2D-MoS_2_ are presented and discussed. In particular, its unique and outstanding optical properties are put forward through theoretical simulations based on the complex permittivity of the MoS_2_ monolayer. In Section 4, density functional theory (DFT) calculations were carried out on both the bulk and the monolayer MoS_2_ using Quantum Expresso™ code and one-dimensional solar cell capacitance simulator SCAPS-1D™. These calculations were used to determine, respectively, the optoelectronic properties and photovoltaic performances in solar cell configuration. Then, interesting applications in three selected fields where 2D-MoS_2_ has shown promising outcomes, namely solar energy conversion, gas sensing, and plasmonics, are presented in Section 5. In the last section, we discuss the reported works and point towards new directions and applications in which 2D-MoS_2_ would potentially play a key technological role.

## 2. Fabrication Techniques of 2D-MoS_2_

Tremendous efforts have been devoted to the synthesis of 2D-MoS_2_ with controllable large-area growth and uniform atomic layers using both top-down and bottom-up approaches. The most commonly used processing routes are detailed in the following sub-sections along with their advantages and limitations.

### 2.1. Mechanical and Chemical Exfoliations

Mechanical exfoliation, also known as micromechanical cleavage, is a straightforward technique that takes advantage of the weak bonding between layers, for the production of high-quality mono- to few-layer MoS_2_ [58,59,60]. It consists of exfoliating thin films of 2D-MoS_2_ from a bulk MoS_2_ crystal by using a low surface tension tape to break the weak interlayer bonds in a similar way as for grapheme [61]. Additional exfoliation of the extracted films may be needed to obtain few- to monolayer MoS_2_. Tapes could be attached to glass slides to achieve planar exfoliation and slow peeling. The obtained monolayers are usually transferred to an appropriate substrate for further analysis and testing.

The advantage of the mechanical exfoliation process lies in its simplicity that requires the sole use of a confocal microscope to localize the 2D-MoS_2_ layers deposited on the substrate. Conveniently, this technique can produce high crystalline quality mono- to few layers with a lateral size up to few tens of micrometers, making them highly suitable for sensing applications. However, this approach suffers from a lack of a consistent control in producing the 2D monolayers as it is heavily user-dependent and does not permit the control of the size and/or thickness uniformity of the exfoliated 2D-MoS_2_ layers [62]. Therefore, the mechanical exfoliation technique is not necessarily suitable for the production of 2D-MoS_2_ layers intended for large-area and high-throughput applications.

Chemical exfoliation, on the other hand, appears as a promising approach to produce large quantities of mono- and few-layer MoS_2_ nanosheets [60,63,64,65]. Eda et al. [54] reported a high yield of monolayer crystal synthesis using chemical exfoliation of bulk MoS_2_ via Li intercalation. However, this approach may induce an alteration in the quality of the produced 2D-MoS_2_. For instance, the chemically exfoliated MoS_2_ layers can lose their semiconducting properties because of the structural changes resulting from the Li intercalation process. However, this fabrication route stands by its ease of processing, low production costs, and suitability for catalysis and/or sensing applications [66].

### 2.2. Chemical Vapor Deposition

**Figure 2:**
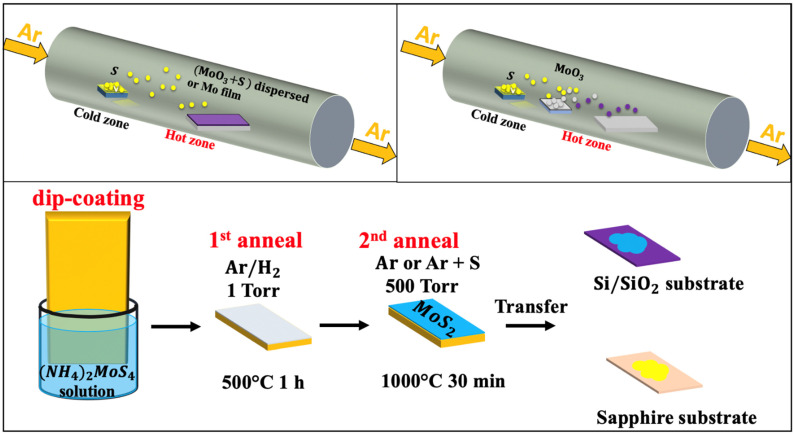
Schematic of the chemical vapor deposition techniques: (**a**) thermal vapor sulfurization process using a quartz tube; (**b**) thermal vapor deposition process using a quartz tube; and (**c**) thermal decomposition of (NH_4_)_2_MoS_4_ (reproduced and adapted from Ref. [34]).

Chemical vapor deposition (CVD) is one of the most popular routes for large-scale, high-quality, and low-cost 2D-MoS_2_ material production [49,67,68,69]. CVD is a bottom-up fabrication method at the equilibrium state, which enables the processing of layered 2D-MoS_2_ with controlled morphology and good crystallinity while minimizing structural defects. The control of the CVD process is ensured by tuning the deposition parameters such as temperature, pressure, gas flow rate, precursor’s quantities, and substrate types. The 2D-MoS_2_ synthesis via the CVD technique can be achieved by means of thermal vapor sulfurization (TVS), thermal vapor deposition (TVD), and thermal decomposition (TD). Deokar et al. [43] used TVS for high quality and vertically-aligned luminescent MoS_2_ nanosheets. A similar process could be used to grow 2D-MoS_2_ layers [36,70] by employing two sources, such as molybdenum thin film (below 20 nm) or molybdenum oxide (MoO_3_) powder deposited on a SiO_2_/Si substrate as a first precursor and the sulfur powder or gaseous sulfur source (H_2_S, etc.) as the second precursor [49,67,68,69,71,72]. A typical CVD sulfurization process (Figure 2a) is usually performed in a tubular furnace reactor, where a continuous argon flow (typical flow rate 100 sccm) is used as a carrier gas to stream the evaporated sulfur into the Mo source materials.

One of the critical aspects to be controlled in such a CVD tubular reactor is the temperature gradient between the S powder and the substrate. In fact, while the S powder is at 150–200 °C, the substrate’s temperature—with or without Mo thin film—should be maintained in the 700–900 °C range to obtain the 2D-MoS_2_ phase. This technique offers sufficient latitude to fairly control the thickness and the homogeneity of the grown 2D-MoS_2_. The typical average lateral crystal size obtained by CVD is usually in the 10–30 nm range. Table 2 shows few examples of CVD-TVS grown MoS_2_ nanostructures along with their associated processing conditions.

Table 2 shows the typical morphologies obtained for MoS_2_, which seem to depend on the carrier gas and the type of the substrate used. The reaction time and the spatial position of the substrate strongly affect the number of resulting layers.

The TVD based MoS_2_ growth (Figure 2b) involves the concomitant evaporation of both MoO_3_ and S powders. This approach consists of a stepwise sulfurization of MoO_3_ to form the MoS_2_ phase. It has been shown that, by increasing the S vapor flux, the sulfurization proceeds through several phase changes before reaching the final product. First, MoO_3_ is formed, then MoO_2_ followed by MoOS_2_, and finally MoS_2_. This approach is very useful to obtain 2D MoS_2_ layers with a lateral size of few tens of microns. The TVD growth conditions of MoS_2_ under various conditions and with different characteristics are summarized in Table 3.

In comparison to the results obtained by CVD-TVS summarized in Table 2, TVD exhibits high-yield fabrication of 2D-MoS_2_ monolayers generally exhibiting a triangular flakes shape. Besides, one can notice the two possible configurations of the substrate of interest in TVD face-up and face-down compared to CVD-TVS [75,76,77,78,79].

Moreover, the TD-based CVD method presents an alternative approach to produce highly crystalline MoS_2_ thin layers with superior electrical properties on insulating substrates [34]. Typically, the TD-CVD is based on the high-temperature annealing of a thermally decomposed ammonium thiomolybdate layer (NH_4_)_2_MoS_4_ in the presence of S, as illustrated in Figure 2c. It is worth noting that the excess in sulfur introduces changes in the shape, size, and morphology of fabricated MoS_2_. It also leads to a p-type MoS_2_ semiconductor by increasing the electrons deficiency. In contrast, the presence of sulfur vacancies in MoS_2_ was reported to have a direct impact on the catalytic properties of MoS_2_, suggesting a carriers’ mobility alteration [80]

Besides, the addition of S during the high-temperature annealing drastically enhances the crystallinity of MoS_2_. Relatively, centimeter-sized MoS_2_ crystals could be formed on Al_2_O_3_ substrates compared to SiO_2_ ones [35]. The fully covered Al_2_O_3_ substrate with an epitaxial monolayer of MoS_2_ was achieved at 930 °C. The MoS_2_ crystals nucleate in a single domain to pursue by domain-to-domain stitching process occurring during annealing at 1000 °C mediated by the oxygen flow. The difference in the self-limited monolayer growth observed between the SiO_2_ and Al_2_O_3_ substrates is related to the absorption energy barrier on MoS_2_ [37]. In particular, the growth of MoS_2_ on Al_2_O_3_ obeys the surface-limited epitaxial growth mode, which is not the case for the SiO_2_ due to lattice mismatch. Moreover, the patterning of the as-grown MoS_2_ layers has been reported by means of the polydimethylsiloxane (PDMS) stamps and the reuse of the substrate after transferring the MoS_2_ layers [35]. Recently, the epitaxial growth of centimeter wafer-scale single-crystal MoS_2_ monolayers on vicinal Au (111) thin films were also obtained at a processing temperature of 720 °C, by melting and re-solidifying commercial Au foils [36]. This allows overcoming the evolution of antiparallel domains and twin boundaries, leading to the formation of polycrystalline films. It has been proposed that the step edge of Au (111) induced the unidirectional nucleation, growth, and subsequent merging of MoS_2_ monolayer domains into single-crystalline films.

### 2.3. Atomic Layer Deposition

The atomic layer deposition (ALD) technique is known to produce high-quality thin films even at low temperatures, typically between 150 and 350 °C. Since ALD is an atom stepwise growth process, where the reactants are alternately injected into the growth area, it allows the purging of excess species and by-products after each reaction. As a result, high-quality films are obtained by sequential surface reactions. A schematic representation of the ALD synthesis of 2D-MoS_2_ can be found elsewhere [81].

Despite the challenges related to its synthesis conditions, ALD makes it possible to deposit crystalline MoS_2_ thin films at a relatively low temperature (<350 °C) followed by annealing. For instance, L.K. Tan et al. [82] reported the possibility to use ALD for the synthesis of highly crystallized MoS_2_ films on sapphire substrates at 300 °C. They prepared MoS_2_ films by alternating exposure of the substrate to Mo(V) chlorides (MoCl_5_) and hydrogen disulfide (H_2_S) vapors. Similarly, Mattinen et al. [83] proposed the use of a Mo based precursor, namely Mo(thd)_3_ (thd = 2,2,6,6 tetramethylheptane 3,5-dionato), with H_2_S as a sulfur source. They have been able to achieve a self-limiting growth and a linear film thickness control (with a very low growth rate of ≈0.025 Å per cycle). While the crystallinity of these MoS_2_ films was found to be particularly good (taking into account that the deposition was done at a low temperature), their surface was rather rough, consisting of flake-like grains with a size of ≈10–30 nm. One of the advantages of this process is the possibility to deposit layered MoS_2_ films on various substrates. Table 4 summarizes the main processing conditions used by different groups along with the achieved MoS_2_ film thicknesses.

The ALD appears as a potentially interesting technique for the production of high-quality MoS_2_ ultrathin films at relatively low temperatures and with the ability to achieve excellent step coverage onto different substrates. However, the very low throughput of the ALD might hinder its scalability and competitiveness in comparison with other physical and/or chemical deposition methods.

### 2.4. Pulsed Laser Deposition

**Figure 3:**
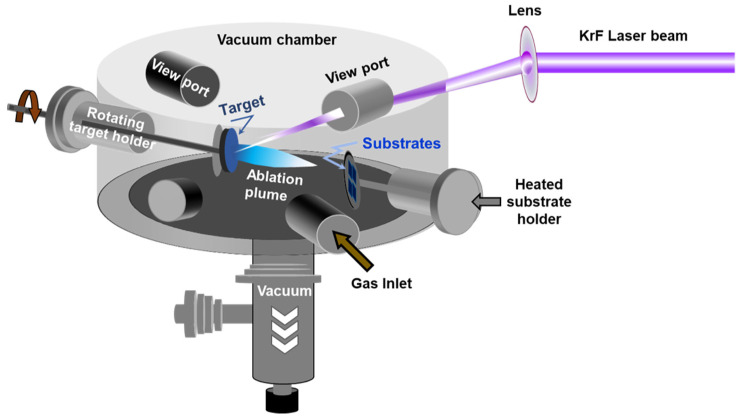
Schematic of the pulsed laser deposition chamber.

Pulsed laser deposition (PLD) has emerged as one of the most promising physical vapor deposition (PVD) techniques for the deposition of MoS_2_ thin films. The PLD approach consists of shining a focused high-power laser beam onto the surface of a solid target to be ablated and deposited as a film on a substrate. PLD is a non-equilibrium process that leads to the absorption of very-short (15–20 ns) and highly-energetic laser pulses by the target and to the formation of a directive plasma plume. The laser-ablated species that form the plasma plume condense onto the substrate, leading to the growth of a thin film. The PLD is well known for its large process latitude, high-flexibility, and excellent process controllability. For instance, by controlling the number of laser ablation pulses and/or the background gas pressure, nanoparticles, and/or films with thicknesses varying from few nm to few microns can be synthesized. Figure 3 shows a schematic representation of a PLD system.

Among the advantages and the unique features of the PLD method, we can cite: (i) its ability to achieve a congruent transfer to the films when a multi-element target is used [91]; (ii) its highest instantaneous deposition rate along with the highly-energetic aspect of the ablated species (~10 times higher than in sputtering) enables the growth of metastable phases and/or crystalline phases even at room temperature; and (iii) its process latitude, which makes it easy to control almost independently each of the deposition parameters (laser intensity, number of laser ablation pulses, background gas pressure, and substrate temperature), and hence the properties of the deposited materials [92,93,94]. While the early studies on the PLD of MoS_2_ date back to the 1990s [95,96,97,98,99,100], it is only recently that important advancements have been made in PLD synthesis of 2D-MoS_2_ films onto various substrates opening thereby the way to their use for different optoelectronic applications. In 2014, PLD was successfully used to grow one to several layers of MoS_2_ onto different metal, semiconducting, and sapphire substrates [101,102]. Siegel et al. [103] were the first to report, in 2015, the growth of MoS_2_ films (from 1 to a few 10s of monolayers thick) on centimeter-sized areas. Other attempts were made to deposit ultrathin (≤3 nm) films of nearly-stoichiometric amorphous MoS_2_ onto irregular surfaces such as silicon and tungsten tips and to study their field electron emission (FEE) properties [95]. The authors stated that the addition of the MoS_2_ coating is beneficial to the FEE process since lower electric fields were required to extract an electron current density of 10 μA/cm^2^ (namely, 2.8 V/μm for MoS_2_-coated Si and ~5.5 V/μm for MoS_2_-coated W tips). More recently, PLD has been used to fabricate high-quality MoS_2_ films (monolayer to few layers) and integrated them into functional ultraviolet (UV) photodetectors [104]. The developed photodetectors were found to exhibit a very low dark current (~10 × 10^−10^ A), low operating voltage (2 V), and good response time (32 ms). Their performance surpassed that previously reported for 2D-MoS_2_ synthesized by other routes [105,106,107,108,109]. Indeed, under UV irradiation, their detectivity, photoresponse (I_on_/I_off_ ratio), and responsivity were found to be as high as 1.81 × 10^14^ Jones, 1.37 × 10^5^, and 3 × 10^4^ A/W, respectively. Table 5 summarizes most of the papers reported so far on the PLD of MoS_2_ films. More specifically, it compares the main PLD growth conditions of 2D-MoS_2_ films along with the obtained crystallographic phase and some of the reported optoelectronic properties.

### 2.5. Other Processing Routes

In addition to the main fabrication methods presented above, other PVD techniques have been used to deposit 2D-MoS_2_ films. Among these methods, magnetron sputtering has been used to deposit both MoS_2_ and WS_2_ films onto polydimethylsiloxane (PDMS) polymer substrates [37,127,128,129,130] with controllable defect densities. The PDMS substrate was chosen to fabricate flexible devices based on 2D-semiconducting materials. Interestingly, very smooth MoS_2_ surfaces, with a roughness of less than 2 nm, were achieved by casting the polymer on a polished silicon wafer. It has also been shown that it is possible to induce subsequent crystallization of MoS_2_ by exposing it to a pulsed 532 nm laser [127].

Finally, the use of any of the above-discussed techniques to fabricate 2D-MoS_2_ films is mostly dictated by the availability of the equipment, expertise, and requirements of targeted application. In a general context, the physical-chemical and optoelectronic properties of the final MoS_2_ films will be determined to select the appropriate synthesis route. Nevertheless, the level of complexity, throughput, and fabrication costs have to be considered to choose the appropriate synthesis technique particularly when a technology has to be adopted. Table 6 provides a general comparison of the preparation techniques of MoS_2_ described in this review by listing their main advantages and limitations.

## 3. Characterizations of MoS_2_ Thin Films

**Figure 4:**
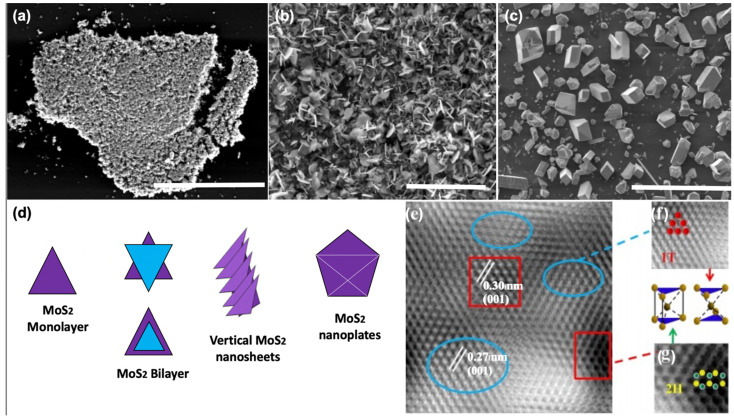
Examples of MoS_2_ microstructures: (**a**) planar triangle flakes scale = 40 µm; (**b**) vertical nanosheets scale = 100 um; (**c**) vertical nanoplates scale = 100 µm; (**d**) schematic of the nucleation process of MoS_2_; (**e**) HRTEM image of mixed 1T-MoS_2_ and 2H-MoS_2_; (**f**) zoom in of blue circled region of the 1T-MoS_2_ structure, with the unit cell of the 1T phase; and (**g**) zoom in of red circled region of the 2H-MoS_2_ structure, with the unit cell of the 2H phase. (Figure 4e–g adapted from Ref. [131] Copyright 2019, Springer Nature.)

To assess the crystalline quality, microstructure, and optoelectronic properties of the synthesized 2D-MoS_2_, a variety of characterization techniques have been employed and reported in the literature. These include optical microscopy (OM), scanning electron microscopy (SEM), high-resolution transmission and Scanning transmission electron microscopy (HRTEM and HRSTEM), atomic force microscopy (AFM), energy-dispersive X-ray spectroscopy, X-ray photoelectron spectroscopy (XPS), Raman spectroscopy, and photoluminescence (PL). These methods are often used to investigate the overall 2D-MoS_2_ surface topography and to qualify the nature of the synthesized material and the shapes of its building blocks (i.e., triangle, nanosheets, and nanoplates) (Figure 4). The observations made by imaging methods are also essential to envision a possible growth mechanism of the micro/nanostructures with respect to the used processing parameters. For instance, Figure 4d shows a schematic representation of the nucleation process of some morphologies of 2D-MoS_2_.

Subsequently, HRTEM investigations could be carried out to precisely characterize the MoS_2_ crystalline structure and examine locally its lattice parameters and the presence of defects. In particular, the HRTEM image depicted in Figure 4e is of great importance, as it was recorded in cross-region containing the two possible crystal configurations of MoS_2_. As it can be seen in Figure 4e–g, the identified phase mixture of 1T@2H-MoS_2_ could coexist simultaneously in the same fabricated MoS_2_ thin film [131].

**Figure 5:**
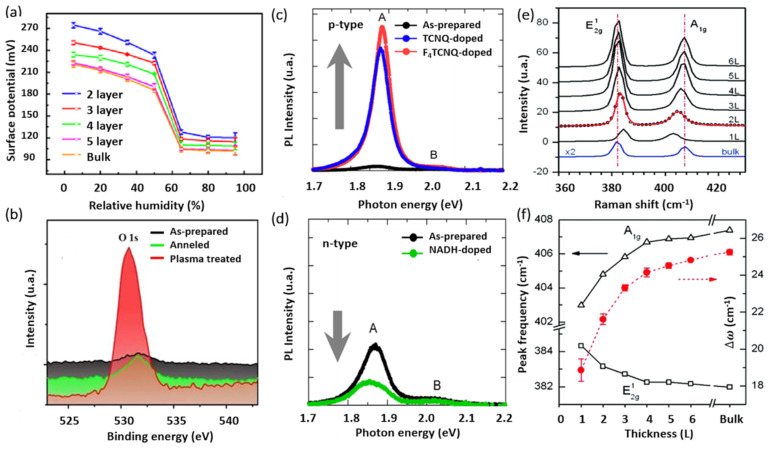
(**a**) Surface potential captured by KPFM vs. relative humidity RHs with respect of the number of MoS_2_ layers (reproduced and adapted from Ref. [133], Copyright 2017, IOP Publishing); (**b**) XPS spectra of Mo 3d and S 2s core levels for different treatment conditions (adapted from Ref. [132] Copyright 2014, American Chemical Society); (**c**,**d**) PL spectra of monolayer MoS_2_ before and after being doped (reproduced from Ref. [134] Copyright 2013, American Chemical Society); and (**e**,**f**) Raman spectra for various MoS_2_ films with respect to the number of MoS_2_ layers (reproduced from Ref. [135] Copyright 2010, American Chemical Society).

AFM and its variant methods constitute key characterization tools for the investigation of 2D crystals, mainly due to the atomically thin nature of this layered class of materials. Both vertical and lateral resolutions are fundamentally required to properly investigate the intrinsic properties of 2D materials. AFM is among the few techniques that allow the characterization of 2D-MoS_2_ in ambient and controlled environments at the nanometer scale. In addition to measuring the local thickness and surface topography, AFM-based electrical methods provide access to additional interesting properties such as the local variations in surface potential of 2D-MoS_2_. For instance, the Kelvin probe force microscopy (KPFM) method allows the characterization of the sample’s surface work function variations. The work function is an extreme surface property, which depends on the energy differences between the Fermi and vacuum levels at the surface. This renders the use of KPFM for the characterization of 2D-MoS_2_ fundamentally important to investigate band alignments in nanostructures and to study the dependencies of local electronic properties on the number of 2D-MoS_2_ layers. It also provides key insights into the environmental effects on the state of the sample surface both electronically and morphologically. The KPFM technique was used (Figure 5a) to determine the surface potential variations in mono- and multilayer MoS_2_, under different humidity conditions.

X-ray photoelectron spectroscopy (XPS) is another relevant surface characterization technique that is widely used to achieve the elemental surface composition of MoS_2_ films as well as their chemical bonding states. Figure 5b shows typical high-resolution XPS spectra of the Mo_3d_ and S_2p_ core levels. The Mo_3d_ region exhibits two characteristic emission peaks at 232.5 (Mo 3d_3/2_) and 229.4 (Mo 3d_5/2_) eV. These binding energy values are consistent with electrons of Mo^4++^ corresponding to MoS_2_. Likewise, the S 2p_3/2_ and S 2p_1/2_ doublet appearing at binding energies of 162.3 and 163.5 eV is typical for S^2-^ in MoS_2_ structure. Nan et al. [132] used XPS to show the PL enhancement of monolayer MoS_2_ through defect engineering and oxygen bonding. The chemical adsorption of oxygen created a heavy p-type doping and the conversion of the Trion into Excitons. Moreover, it caused the suppression of the non-radiative recombination of the excitons at the defect sites. Their results were verified by PL measurements at low temperature, as shown in Figure 5c,d.

Unlike bulk MoS_2_, the ultrathin 2D-MoS_2_ (i.e., one to few layers) exhibits a strong PL intensity which increases with reducing the number of layers [136], which has been attributed to quantum confinement effects [53,137]. The PL response can be tuned via several mechanisms including doping [134], plasmonic effect, and defects engineering [132]. For instance, Mouri et al. [134] studied the influence of the thickness on the PL response of MoS_2_ by using mono-, bi-, and trilayer MoS_2_ and the PL modulation using doping. They demonstrated that p-type doping with high electron affinity seems to enhance the PL intensity, while the n-type doping tends to reduce it, as illustrated in Figure 5c,d.

Moreover, Raman spectroscopy presents a very sensitive, fast, and non-destructive technique to access valuable information on the chemical structure, phase and polymorphs, crystallinity, and chemical bonding states of 2D-MoS_2_ materials. It allows the monitoring of the two characteristic peaks of MoS_2_, namely the in-plane and out-of-plane vibration modes E^1^_2g_ and A^1^_g_ appearing for 514 nm excitation energy at the respective positions of 384.5 and 404.6 cm^−1^ for 2D-MoS_2_ monolayer [135] (Figure 5e). More interestingly, the difference between the peak positions of E^1^_2g_, A^1^_g_ (Δω) can be used as a robust and effective diagnostic to determine the number of MoS_2_ layers (up to four layers) or to simply estimate the MoS_2_ film thickness (Figure 5f). Usually, Δω is less than 20 cm^−1^ in the presence of a single layer of MoS_2_, but it increases with increasing MoS_2_ thickness to reach 25 cm^−1^ for the bulk MoS_2_ [135]. In fact, a thorough study on the dependence of the characteristic Raman peak positions, width, and intensity of MoS_2_ films on their thickness have been investigated [103,135,138]. Furthermore, H. Li et al. [138] reported that the frequency of the characteristic peaks is strongly dependent on the excitation energy due to the resonance effect. They showed a red shift of the E^1^_2g_ mode of about 2.2 cm^−1^ and blue shift of the A^1^_g_ mode of about 4.1 cm^−1^. Thus, to effectively determine the exact MoS_2_ number of layers using Raman spectroscopy, one has to consider the excitation energy and the thickness limit at which the Raman vibrations frequency is reaching a plateau, indicating that it is less sensitive to MoS_2_ thickness variation above four layers.

## 4. Band Structures and Electronic Properties

**Figure 6:**
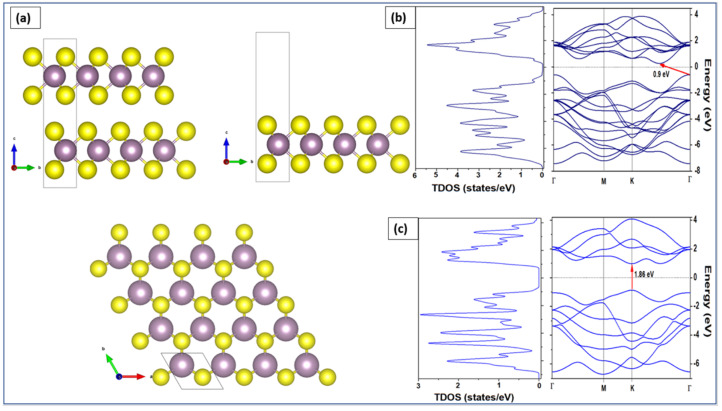
(**a**) Bulk MoS_2_ (**top-left**), monolayer MoS_2_ (**top-right**), and top view of MoS_2_ monolayer (**bottom**). Total density of states (**left**) and band structure (**right**) of the (**b**) bulk and (**c**) monolayer.

We employed density functional theory (DFT) to determine the optoelectronic properties in particular the bandgap energy of both bulk and monolayer MoS_2_. Perdew–Burke–Ernzerhof (PBE) approach was applied to describe the electronic states of MoS_2_ using band structure and the density of states (DOS). DFT calculations were implemented in Quantum Espresso™ code [139,140]. The considered 2H-MoS_2_ has a hexagonal crystal form with the space group P63/mmc (No. 194). The equivalent positions for this structure employed in the calculations are Mo (1/3, 2/3, and 2/8) and S (1/3, 2/3, and 0.621). The valence electron configuration selected for Mo and S atoms are 4p^5^ 5s^1^ and 3s^2^ 3p^4^, respectively. The cutoff wave function and the cutoff charge densities are 70 and 700 Ryd, respectively [140]. The cell parameters and atomic positions were fully relaxed by the process of the total energy minimization. The values of the relaxed lattice constants for bulk MoS_2_ are a = 3.15 Å and c = 12.3 Å, respectively. The optimized structure was used to perform calculations for band structures and the total density of states for both MoS_2_ bulk and monolayer. For bulk MoS_2_ (top left panel of Figure 6a), 9 × 9 ×2 k-points were used to obtain the band structure along the path Γ-K-M-Γ in the Brillouin zone. For MoS_2_ monolayer (top right panel of Figure 6a), 9 × 9 × 1 k-points were used. A 15 Å vacuum along the z-axis above the monolayer was added to isolate the MoS_2_ and prevent any interaction between the adjacent layers [141]. The top view of the MoS_2_ monolayer is shown in the bottom panel of Figure 6a, where sulfur atoms are represented in yellow and molybdenum atoms are shown in purple.

To obtain the electronic properties, the MoS_2_ bulk was considered as a set of two hexagonal planes linked together by weak Van Der Waals bonds. The MoS_2_ monolayer was considered as a single hexagonal plane with covalent bonds between atoms S-Mo-S [142]. The left panel of Figure 6b shows the total DOS calculation results of the bulk MoS_2_ while the right panel of Figure 6b shows the calculation of its band structure. The energy range is between −8 and 4 eV versus the directions of the highest symmetries in the first Brillouin zone Γ, M, K, and Γ. As observed from the band structure calculations, the MoS_2_ bulk has an indirect bandgap of 0.9 eV. The minimum of the conduction band is located between K and G and the maximum of valence band at point G. This indirect bandgap obtained for the MoS_2_ bulk was attributed to the presence of interlayer interactions in the bulk structure [143]. In contrast, Figure 6c shows that the monolayer MoS_2_ has a direct bandgap of 1.89 eV at the K point. The DOS results are compatible with the results of the band structure. Similar conclusions have been stated in other investigations [141,142].

## 5. MoS_2_ Applications

Because of their attractive optoelectronic properties, possibly tunable by for example controlling the number of monolayers, MoS_2_ thin films were tested and validated for a variety of applications including electronics, photonics, solar energy, and energy storage. Here, we give a few examples of some specific successful and promising applications of MoS_2_ films for solar energy conversion [144,145], gas sensing [44,48,146,147], and plasmonics [148,149,150,151,152].

### 5.1. MoS_2_ for Solar Energy Harvesting

As demonstrated by DFT calculations, 2D-MoS_2_ exhibits interesting optoelectronic properties attributed to its direct bandgap ranging from 1.2 to 1.9 eV and an absorption coefficient greater than 105 cm^−1^ throughout the solar spectrum. These key properties are very promising for the use of MoS_2_ in photovoltaic (PV) applications. Indeed, it has been shown that, when a monolayer of n-type MoS_2_ is deposited onto a p-type silicon substrate, the resulting p-n junction based PV device is able to yield a power conversion efficiency (PCE) as high as 5.23%, as recorded elsewhere [153]. Such a PV performance is most likely a consequence of the excellent ability of MoS_2_ to efficiently separate the generated photo-charges at the n-MoS2/p-Si interface of the heterojunction.

**Figure 7:**
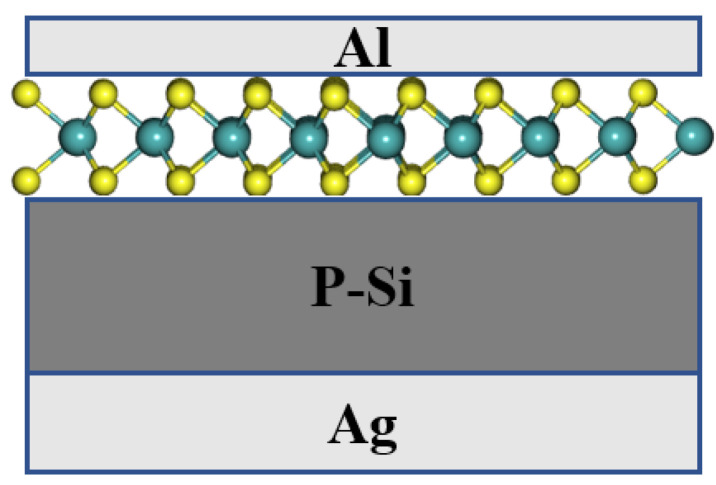
Simulated solar cell structure of solar cell.

To highlight the electrical performance of thin films MoS_2_-based solar cells in a homojunction form, we used the one-dimensional solar cell capacitance simulator SCAPS-1D™ software 3.3.08 interface [154], developed by M. Burgelman – Department of Electronics and Information Systems at the University of Ghent, Belgium [155,156], to calculate the different PV parameters, i.e., open circuit voltage V_OC_, short-circuit current density J_SC_, fill factor FF, and PCE (η). In this sense, a solar cell made of Ag/p-Si/MoS_2_/Al structure, as the one represented by a schematic in Figure 7, was implemented in the SCAPS-3308™ environment.

The simulations were made under AM1.5 illumination conditions at an operating temperature of 300 K. The physical parameters related to the electronic properties of the layers used in the simulation are shown in Table 7. For the considered junction, the thermal speed of the electrons and the holes were fixed at 10^7^ cm/s, the type of defect is neutral, and the capture cross section is 10^−14^ cm^2^.

Beyond, the input parameters used in our SCAPS simulations, we provide hereinafter a survey of commonly used physical parameters of MoS_2_ reported in the literature to simulate the performance of MoS_2_ in PV applications. As can be seen in Table 8, several combinations are possible which may yield different results.

**Figure 8:**
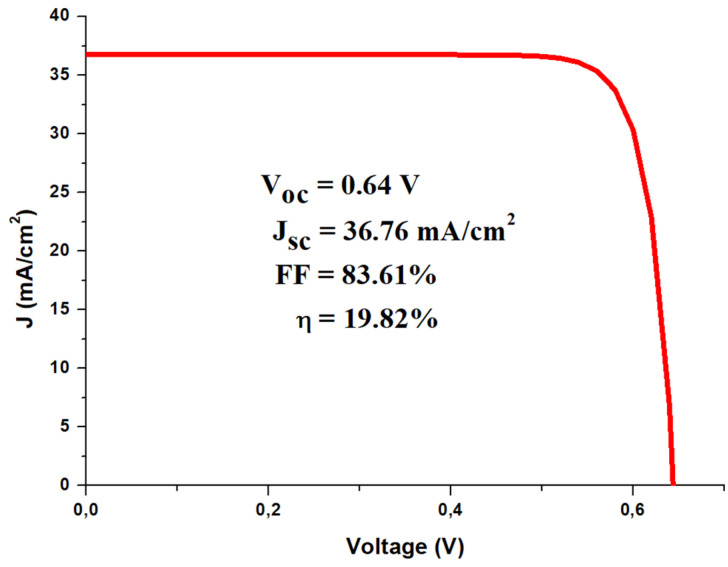
Simulated J-V characteristic of p-Si/n-MoS_2_ solar cell, as calculated by SCAPS-1D™ software.

The outcome of our simulations shows that the p-Si/n-MoS_2_ structure in Figure 7 can yield a PCE value as high as 19.82% when considering 2D-MoS_2_ with the highest bandgap of 1.9 eV. Figure 8 shows the simulated J-V curve of the p-Si/n-MoS_2_ cell along with its associated PV parameters. The rather high V_oc_ value of 0.64 V reflects the strong built-in electrical field at the interface between the n-MoS_2_ layer and p-Si substrate.

The high PCE obtained is comparable to the one obtained for well-proven solar cell materials. This is an outstanding yield for an only 0.33 nm thick material used in conjunction with p-Si in the solar cell set up as compared to 250 µm thickness used for conventional Si technology. Moreover, sulfur and molybdenum are abundant and cheaper raw materials as compared to the technologies achieving similar performances such as III-V materials.

Nevertheless, although the simulated PCE performance underlines the great potential of 2D-MoS_2_ films for PV devices, other challenging issues still need to be addressed or mitigated to develop such devices. For instance, the controlled deposition of MoS_2_ monolayer, the achievement of a reliable metal contact on MoS_2_ monolayer free of leakage current or a shortcut with the underlying Si substrate, and the scalability of 2D-MoS2 ultrathin films to the well-established large-size Si wafer technology are among the challenging issues to be addressed in future works.

### 5.2. MoS_2_ for Gas Sensing Applications

MoS_2_ nanosheets (NS) have been reported to exhibit enhanced gas sensing performances for a variety of gases, including toxic and hazardous gases such as ammonia (NH_3_) and nitrogen dioxide (NO_2_) [43,48,146,165,166,167]. Thus, MoS_2_ NS act as a simple chemiresistor that changes its electrical resistance when in contact with reactive gases. The sensing response or sensitivity (S) towards a target gas, at a given operating temperature, is determined from the measured values of resistances of the MoS_2_-NS sensing element in the presence of atmospheric air resistance (R_a_) and target gas (R_g_). Usually, the target gas molecules adsorb onto the MoS_2_ NS exposed edges and changes its conductivity through the donor/acceptor exchanges process. The sensitivity (S) is defined as follows:

$$S=\frac{R_{a}-R_{g}}{R_{g}}$$

To design an effective 2D-MoS_2_ gas sensor, care must be taken to the optimization of its operating temperature, response/recovery times, and selectivity. 2D-MoS_2_-based gas sensors were found to offer certain advantages, such as high-temperature stability, high resistance to a corrosive environment, and high sensitivity [26,146,165,166]. In addition, 2D-MoS_2_ thin film-based sensors were reported to detect NH_3_ triethylamine (TEA) molecules at the sub-ppm level, at an operating temperature as low as 30 °C [147].

**Figure 9:**
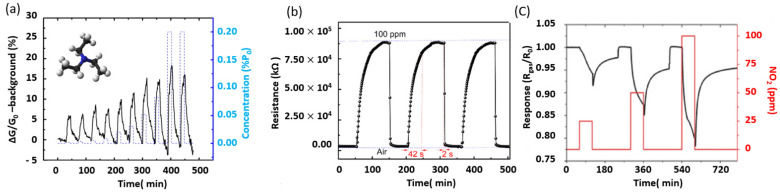
(**a**) MoS_2_ sensor response exposed to TEA (adapted from Ref. [147] Copyright 2013, American Chemical Society); (**b**) repeatability and reversibility of the FLMN gas sensor at 100 ppm NO_2_ concentration (reproduced and adapted from Ref. [168], Copyright 2019, MDPI); and (**c**) the MoS_2_/CNT sensor response as a function of three NO_2_ concentrations (25, 50, and 100 ppm) (reproduced and adapted from Ref. [42], Copyright 2017, Wiley-VCH).

MoS_2_ thin films obtained by mechanical exfoliation were used for highly sensitive field-effect transistor (FET) sensors [147]. By varying the number of MoS_2_ layers, the MoS_2_-based FET sensor exhibited high nitrogen monoxide (NO) sensitivity with a detection limit of 0.8 ppm. Moreover, DFT calculations indicated that NO and NO_2_ seemed to strongly bind to MoS_2_ nanosheets in contrast to other molecules such as carbon monoxide (CO), carbon dioxide (CO_2_), NH_3_, NO, NO_2_, and CH_4_. In addition, the exfoliated MoS_2_ monolayer showed high response to triethylamine (TEA) at concentrations ranging from 1 to 100 ppm at room temperature (Figure 9a). Due to the strong response and excellent signal-to-noise ratio, a detection limit of TEA as low as 10 ppb was achieved.

Furthermore, exfoliated few-layer MoS_2_ nanosheets deposited on a substrate with interdigitated electrodes demonstrated good NO_2_ detection performances at room temperature [168]. The reported device shows a quick and complete recovery time of 2 s at a rate greater than 97%. Similarly (Figure 9b), DFT calculations indicated that the fairly fast recovery of MoS_2_ arises from the weak van der Waals interactions between NO_2_ and the MoS_2_ surface.

It is worth mentioning that, regardless of their form or morphology, MoS_2_ thin films remain as robust gas sensors. Indeed, atomic layered MoS_2_ fabricated by CVD showed excellent sensitivity and high selectivity once exposed to NH_3_ and NO_2_ [169]. The resistance of the MoS_2_ films increases in the case of NO_2_ adsorption, while it decreases for the NH_3_ adsorption. The recovery rate of NO_2_ is higher at 100 °C than at room temperature, while the NH_3_ sensing signal is negligible at 100 °C. To further exploit the large affinity of NO_2_ with MoS_2_ thin films, MoS_2_ hexagonal-shaped nanoplates (HNPs), with exposed edges allowing significant charge transfer, were grown on the top 20 nm of carbon nanotubes (CNTs). This configuration is advantageous to increase both the surface area and the number of sites for gas adsorption. The hybridization of MoS_2_ by deposition on CNTs showed an enhanced room-temperature gas-sensing performance [42], attaining a detection limit of a few ppb of NO_2_ concentration.

### 5.3. MoS_2_ for Plasmonic Applications

Because of their optical bandgap spread, MoS_2_ thin films offer interesting opportunities to be coupled with noble metal nanoparticles (NPs) in order to exacerbate the plasmonic properties. Indeed, the coupling effects between the excitons from MoS_2_ with the plasmons generated within the metal NPs open various prospects for tunable light emitters and absorbers over a wide spectrum. Various MoS_2_-related plasmonic structures have been developed for different optoelectronic applications, including photodetection [152], photoluminescence modulation [150], photocatalysis [170,171], and photovoltaics [172].

**Figure 10:**
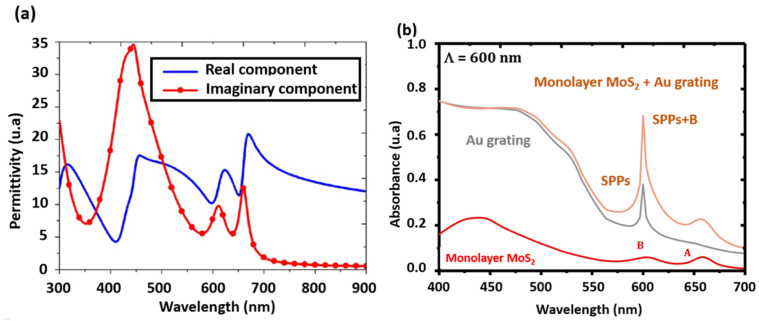
(**a**) Complex permittivity vs. wavelength of monolayer MoS_2_ (reproduced and adapted from [174], Copyright 2018, The Optical Society, OSA); and (**b**) spectral absorptances of an opaque Au plate, a simple 1D Au grating, a suspended monolayer MoS_2_, and monolayer MoS_2_-covered Au grating for TM waves with normal incidence from 400–700 nm (reproduced from Ref. [175] Copyright 2018, Elsevier).

To better understand the origin of the enhancement in light emission/absorption properties of MoS_2_/metal-NPs hybrid structures, it is necessary to comprehend and estimate the variation of 2D-MoS_2_ complex permittivity. A mathematical approach based on hybrid Lorentz–Drude–Gaussian (HLDG) model was proposed by Mukherjee et al. [173] to describe the complex permittivity of MoS_2_ monolayer based on its absorption spectrum (Figure 10a).

The HLDG model can be presented as follows:

$$\varepsilon_{c}=\varepsilon_{c}^{LD}+\varepsilon_{c}^{G},$$

where the superscripts LD and G correspond to Lorentz–Drude and Gaussian permittivity terms, respectively, as described elsewhere [173].

Chen et al. [176] used the HLDG model to design and simulate a perfect absorber based on the local surface plasmon resonance (LSPR) and the coupling properties between Ag patterns and a MoS_2_ monolayer. Their results show that MoS_2_ could increase the optical absorption dramatically. In another work, Jiang et al. [174] integrated the generalized interference theory in the HLDG model to investigate the optical properties of a broadband absorber utilizing a MoS_2_ monolayer. A more rigorous approach, consisting in the use of a coupled-wave analysis algorithm with the HLDG model, has been proposed to study the optical absorption of a composite photonic structure made of MoS_2_ Au grating [175]. The authors showed that the optical absorption of Au grating can be strongly modified by altering the number of MoS_2_ layers (Figure 10b), changing the layout of the MoS_2_ layer (e.g., to a MoS_2_ nanoribbon array), or inserting a hafnium dioxide spacer. Furthermore, they showed an enhancement of the localized electromagnetic field due to surface plasmon polaritons triggered by Au grating in the presence of few layers of MoS_2_. The observed enhancement of the MoS_2_ optical absorption was mainly attributed to the exciton transition. Additionally, the HLDG model was used by Xiaoyong et al. [149] to investigate the tunability of wave propagation in MoS_2_ supported hybrid surface plasmons waveguides based on dielectric fiber-gap metal substrate structures. By using the finite element method, these authors examined the influence of the structural parameters, the dielectric fiber shape and carrier concentration of the MoS_2_ layer on the hybrid modes. Their results allow identifying the tunable parameters of the hybrid modes of waveguide structures that could lead to the design of novel surface plasmon devices in the future.

On the other hand, the association of MoS_2_ with plasmonic NPs was also exploited by Yang et al. [151]. The authors reported on the fabrication of a hybrid nanostructure where a MoS_2_ monolayer is transferred onto the surface of 10-nm-wide Au nanogap arrays. Interestingly, by adjusting the length of the Au nanogaps, the authors achieved a photoluminescence enhancement as high as ~20 folds. In a more recent work, Mawlong et al. [150] also reported a much higher enhancement factor ~463 folds compared to pristine MoS_2_ monolayer at ambient of the PL intensity in the case of TiO_2_/Au/MoS_2_ ternary core–shell hetero-nanostructures. Such a strong PL enhancement was attributed to the heavy p-doping of the MoS_2_ lattice along with LSPR initiated exciton–plasmon coupling at the MoS_2_/Au interface [148]. These results suggest that the hybridization of MoS_2_ with appropriate metal nanostructures enhances the photoresponse. Indeed, Rahmati et al. [152] also reported an enhancement in the photocurrent generated by vertically aligned MoS_2_ nanosheets decorated with Au NPs.

## 6. Summary and Outlook

Based on the ever-increasing number of published works on 2D-TMDs materials, there is no doubt that MoS_2_ will continue to be one of the materials of the choice for the development of innovative and potentially scalable optoelectronic devices.

In term of fabrication, the CVD technique remains a comfortable and affordable route for continuous developments of a variety of shapes and morphologies of 2D-MoS_2_. By gaining more control of the deposition process itself, it is possible to further tune the optical and electrical properties of MoS_2_ nanostructures while increasing the size of the sample and the lateral uniformity. Of particular concern is the need to improve the reproducibility of defect-free structures. On the other hand, PLD appears as a highly promising alternative for the production of high-quality MoS_2_ thin films with a fairly high-level of homogeneity. It also allows tuning the MoS_2_ strain level during the elaboration, which may lead to exotic physical properties. PLD also offers an additional possibility to optimize, almost quasi-independently, different deposition parameters of MoS_2_ films, and hence tune at will their properties of interest. Finally, PLD also has the advantage of growing crystalline 2D-TMDS at room temperature, which opens the way to deposit MoS_2_ films onto flexible and thermo-sensitive substrates, thereby leading to a variety of new applications.

Regarding the applications, apart from those described in this review, 2D-MoS_2_ exhibits very appealing performances in infrared domains especially in combination with metamaterials such as passive radiative cooling. There are some emerging works [177,178,179,180] related to this aspect such as developing hybrid MoS_2_ thin films with new structures, including metamaterials, metasurfaces, photonic crystals, plasmonics, etc. Similarly, the development of 2D material-based antennas remains unsatisfactory as most of the known achievements on MoS_2_ in this domain are developed theoretically. Especially, the recent works [181] on terahertz (THz) plasmonics have shown the potential of MoS_2_ for their application in antenna research. Precisely, the use of MoS_2_ as a conductive medium in THz antenna appears as a potential direction of recent developments.

## Figures and Tables

**Table 1:** Crystal parameters and the nature of polymorphic structures of 2D-MoS_2_.

Polymorphic Structure	Lattice Parameter	Point Group	Electronic Behavior	Ref
1T	a = 5.60 Å, c = 5.99 Å	D_6d_	Metal	[31]
2H	a = 3.15 Å, c = 12.30 Å	D_6h_	Semiconductor	[32]
3R	a = 3.17 Å, c = 18.38 Å.	C_3v_	Semiconductor	[33]

**Table 2:** Examples of CVD-TVS grown MoS_2_ nanostructures.

Substrate	Precursors	Growth Conditions	Morphology	Ref
Si	MoO_3_ and S powders dispersed on substrate	MoO_3_ and S powders dispersed on substrate at 850 °C; S powder at 400 °C; Ar-0.725 L/min; time reaction = 30 min	MoS_2_ nanosheets	[43]
Si [001]	S powder and Mo film deposited on substrate	Mo deposited on Silicon at 850 °C, S at 400 °C; Ar-0.725 L/min; time reaction = 30 min	MoS_2_ nanosheets	[44]
Si/SiO_2_	S powder and Mo film deposited on substrate	Mo deposited on Silicon at 850 °C, S at 400 °C; Ar-0.725 L/min; time reaction = 30 min	MoS_2_ nanosheets	[49]
Diamond substrate	S powder and Mo deposited on substrate	Mo deposited on Silicon with S powder at 800 °C; N_2_; ambient pressure; time reaction = 30 min	Horizontally and vertically MoS_2_	[73]
Si/SiO_2_	S powder and MoO_3_ deposited on substrate	MoO_3_ film deposited on Silicon at 750–850 °C, 600 mg of S powder at 100 °C; Ar-0.01 L/min; time reaction = 10 min	Mono-to few-layers of MoS_2_	[74]

**Table 3:** Examples of TVD grown MoS_2_ along with their relevant processing conditions (* D is the distance between the MoO_3_ and S powders inside the tubular furnace).

Substrate/Setup	MoO_3_ (mg)	S (mg)	D * (cm)	Gas, Flow (sccm)	T (°C), Time (min)	Morphology	Ref
Si face-down	15	80	18	Ar 10 to 500	700, 30	Flake size between 5.1–47.9 µm	[75]
SiO_2_/Si face-up	10	200	30	Ar, 100	850, 20	Monolayer, bilayer and trilayer MoS_2_	[76]
SiO_2_/Si face-down	10	100	–	N_2_, 20	650, 20	MoS_2_ monolayer	[77]
SiO_2_/Si face-down	10-30	–	25	Ar, 150	800, 10	MoS_2_ triangular flakes	[78]
SiO_2_/Si face-up	50	175	–	N_2_, 300	750, 15	MoS_2_ monolayer with lateral size of 50 µm	[79]

**Table 4:** Summary of the ALD deposition conditions and achieved MoS_2_ film thicknesses.

Substrate	Precursors	P (Torr)	T (°C)	Cycles	Thickness	Ref
SiO_2_/Si	Mo hexacarbonyl and dimethyldisulfide	1.4–3.3	100	100	≈11 nm	[84]
SiO_2_/n-Si	MoCl_5_ and H_2_S	0.75	350–450	100	≈9 nm	[85]
Al_2_O_3_	Mo(NMe_2_)_4_ and H_2_S	–	60	100	≈12 nm	[81]
Al_2_O_3_ 2-inch wafer	MoCl_5_ and H_2_S	0.001	300	50	≈9 nm	[82]
SiO_2_/Si	Mo(thd)_3_ (thd = 2,2,6,6 tetramethylheptane 3,5-dionato) and H_2_S	3.75	300	100	≈25 nm	[83]
Al_2_O_3_ c-plane	MoCl_5_ and hexamethyldisilathiane	3.75	350	250	≈22 nm	[86]
Carbon nanotubes, Si-wafers and glass	bis(tbutylimino)bis(dimethylamino) Mo (VI) and H_2_S	300	100–250	100	≈11 nm	[87]
Si, SiO_2_, Al_2_O_3_	MoCl_5_ and H_2_S	3.75	430–480	1	1 layer	[88]
Si	MoCl_5_ and H_2_S	–	390–480	100	≈21.5 nm	[89]
SiO_2_	Mo hexacarbonyl and H_2_S	–	175	100	≈5 nm	[90]

**Table 5:** Summary of the PLD conditions of MoS_2_ films along with their thickness and some of their properties.

Substrate	Target	P(Pa)	T(°C)	Laser Energy	Thickness	Properties	Ref
Stainless steel	MoS_2_	2.66 × 10^−6^	RT/200/300/450	5 mJ	≈400 nm	Granular structure stoichiometric, crystalline MoS_2_	[110]
Stainless steel	MoS_2_	10^−6^	RT/300	100 mJ	≈70 nm	Stoichiometric single crystal MoS_2_	[111]
c-Al_2_O_3_ (0001) and Si/SiO_2_	2H-MoS_2_	9.33 × 10^−4^	600	500 mJ/cm^2^	≈1.4 nm	Stoichiometric 2H phase Flake size ≈ 10 µm	[112]
GaN/c-Al_2_O_3_ (0001)	2H-MoS_2_	8 × 10^−4^	700	50 mJ	Few layers	Mixed phase Roughness ≈0.11 nm	[102]
Titanium foil	p-MoS_2_	1.33 × 10^−2^	RT	–	0.65 nm	1T phase MoS_2_	[113]
SiO_2_ on Si [100]	MoS_2_	1.33 × 10^−2^	800	200 mJ/cm^2^	≈20–60 nm	2H phase MoS_2_	[104]
Gold-coated carbon cloth	Amorphous MoS_2_	1.33 × 10^−2^	RT	220 mJ/cm^2^	≈200 nm	2H phase MoS_2_	[97]
Quartz	MoS_2_	9 × 10^−5^	300	8500 mJ/cm^2^	30 layers	Mixed phase	[114]
Al_2_O_3_ (0001)	MoS_2_+S Powder	1.33 × 10^−2^	700	50 mJ	1–15 Layers of MoS_2_	p-MoS_2_ 2H phase MoS_2_ Roughness of 0.27 nm	[101]
Si	MoS_2_	4 × 10^−4^	RT	5/10/100/400 mJ/cm^2^	≈100–200 nm	Various compositions of MoS_x_ (x ≤ 2.2)	[115]
SiO_2_	MoS_2_	3 × 10^−5^	700	200 mJ	1–5 layers	2H phase MoS_2_	[116]
W (100)-tip	MoS_2_+poly(vinl)	5 × $10^{-3}$	700	2000 mJ/cm^2^	≈20–60 nm	nearly stoichiometric 2H phase MoS_2_	[95]
n-Si and p-Si	MoS_2_+poly(vinl)	5 × $10^{-3}$	700	500 mJ/cm^2^	≈20–60 nm	nearly stoichiometric 2H phase MoS_2_	[95]
Al, Ag, Ni, Cu	MoS_2_	2.6 × 10^−5^	500	50 mJ	≈5 nm	Epitaxial growth of 2H phase MoS_2_	[98]
Sapphire Quartz SiO_2_ HfO_2_	MoS_2_ +S powder	1.33 × 10^−2^	700	30 mJ	1 monolayer—2.8 nm	large-area growth of stoichiometric layered 2H phase MoS_2_	[117]
SiO_2_/Si	MoS_2_	10^−5^	700	200 mJ	few-layer	2H phase MoS_2_	[118]
SiO_2_/Si	MoS_2_ powder	5 × 10^−4^	600	2200 mJ/cm^2^	13 nm	Epitaxial growth of 2H phase MoS_2_	[119]
Si	MoS_2_	10^−4^	RT	100 mJ	129–1900 nm	Stoichiometric films	[120]
c-plane sapphire	MoS_2_	10^−3^	800	2000–3000 mJ/cm^2^	1–5 layers	Epitaxial growth of 2H phase MoS_2_	[121]
Quartz glass	Polycrystalline MoS_2_ powder	5 × 10^−4^	300	8500 mJ/cm^2^	9–10 monolayers	nearly stoichiometric 2H phase MoS_2_	[122]
Quartz	MoS_2_	8.9 × 10^−5^		600 mJ	≈5.8 nm	2H phase MoS_2_	[123]
SiO_2_/Si	MoS_2_@Ag	1.33 × 10^−7^	500	1000–2000 mJ/cm^2^	≈1.3–12.8 nm	2H phase MoS_2_	[124]
fluorophlogopite mica	MoS_2_	10^−5^	700	4000 mJ/cm^2^	≈3.3 nm	2H phase MoS_2_	[125]
Al_2_O_3_ (0001)	MoS_2_	10^−3^	650	100 mJ	≈400 nm	2H phase MoS_2_	[126]

**Table 6:** Comparison of the advantages and limitations of different preparation techniques of MoS_2_.

Techniques	Advantages	Limitations
Mechanical exfoliation	- High-quality and good crystallinity. - Mono- to few-layer MoS_2_ - Simple process	- Long processing time (8–84 h) - Tedious and no controllability - Difficult integration with micro/optoelectronic processing
Chemical exfoliation	- Large-scale growth - Synthesis of MoS_2_ monolayer	- Loss of semiconducting properties of MoS_2_ during Li intercalation.
Chemical vapor deposition	- High-quality and crystallinity - Centimeter-scale area growth - Good control of morphologies	- Caution due to the use of toxic precursors - High synthesis temperatures requirement - No lateral uniformity - Mixed phases of 1T, 2H, etc.
Atomic layer deposition	- Low-temperature deposition - Uniformity of MoS_2_ films - High quality of uniformity - Excellent step coverage	- Very low throughput - Long processing time - High cost
Pulsed laser deposition	- High-quality and faithful transfer of film stoichiometry - Nanometer-level control of the film thickness - Uniformity onto a large surface (up to 3” or 4” diameter wafers) - Quasi-independent control of the growth parameters. - Room-temperature deposition of crystallized MoS_2_ - Compatibility with electronic and optoelectronic device processing	- Relatively costly - Presence of ablated particulates on the surface
Sputtering	- High quality and uniformity onto large surface - Compatibility with electronic and optoelectronic device processing. - Fair thickness control	- Relatively costly - Preferential sputtering - Less control on the stoichiometry

**Table 7:** Physical parameters of n-MoS_2_ monolayer and p-Si substrate used in the SCAPS-1D™ simulations.

Parameters	p-Si [SCAPS]	n-MoS_2_
Thickness (nm)	200	0.32
Bandgap (eV)	1.12	1.9 [153]
Electron affinity (eV)	4.5	4.2 [153]
Dielectric permittivity (relative)	11.9	10.5 [157]
CB effective density of states (1/cm^3^)	2.8 × 10^19^	2.2 × 10^18^ [158]
VB effective density of states (1/cm^3^)	1.04 × 10^19^	1.8 × 10^19^ [158]
Electron thermal velocity (cm/s)	1 × 10^7^	1 × 10^7^ [159]
Hole thermal velocity (cm/s)	1 × 10^7^	1 × 10^7^ [159]
Electron mobility (cm^2^/Vs)	1500	150 [20]
Hole mobility (cm^2^/Vs)	4500	86 [159]
Shallow uniform donor density (1/cm^3^)	0	1 × 10^17^ [159]
Shallow uniform acceptor density NA (1/cm^3^)	1 × 10^16^	0

**Table 8:** A survey of the physical parameters of MoS_2_ used for the simulation of photovoltaic applications.

PV Parameters	Reported Values and References			
Bandgap	1.29 eV [158,160,161]	1.2–1.8 eV [159]	1.23 eV [162]	1.8 eV [163]
Electron affinity	4.2 eV [158,160,161,162,163]	4–4.7 eV [159]	4.22 eV [163]	–
Relative dielectric permittivity	3 [164]	4 [160,161,162]	7 [159]	13.6 [158]
Effective density of states in conduction band	10^16^ cm^−3^ [163]	7.5 × 10^17^ cm^−3^ [160,162]	2.2 × 10^18^ cm^−3^ [158,161]	10^19^, 2.5 × 10^20^ cm^−3^ [159,164]
Effective density of states in valance band	10^17^ cm^−3^ [163]	1.8 × 10^18^ cm [160,162]	~10^19^ cm^−3^ [158,161,164]	2.5 × 10^20^ cm^−3^ [159]
Electron thermal velocity	10^5^ cm/s [162]	2.12 × 10^7^ cm/s	–	–
Hole thermal velocity	10^7^ cm/s [162]	1.18 × 10^7^ cm/s [161]	–	–
Electron mobility	44 cm^2^/Vs [159]	50 cm^2^/Vs [161]	100 cm^2^/Vs [158,160,162]	–
Hole mobility	30 cm^2^/Vs [161]	86 cm^2^/Vs [159]	150 cm^2^/Vs [158,160,162]	
Shallow uniform donor density	10^16^ [161]	10^17^ [164]	10^18^ [162]	–
Shallow uniform acceptor density	10 cm^−3^ [161]	10^17^ cm^−3^ (MoS_2_ type P) [158]	10^21^ cm^−3^ (MoS_2_ type P) [160]	–

## Data Availability

This review contains both data from literature and own authors’ work. The cited data can be consulted in the relevant cited article. The authors data: DFT calculations and SCAPS simulations are available upon request.

## Abbreviations

1T	Tetragonal
2D	Two-dimension
2H	Hexagonal
3R	Rhombohedral
Δω	Raman shift between the peak positions of E^1^_2g_ and A^1^_g_
ALD	Atomic layer deposition
A^1^_g_	MoS_2_ out-of-plane Raman vibration mode
BL	Buffer layer
CNTs	Carbon nanotubes
CVD	Chemical vapor deposition
DFT	Density-functional theory
E^1^_2g_	MoS_2_ in-plane Raman vibration mode
ETL	Electron transport layer
EQE	External quantum efficiency
FEE	Field electron emission
FET	Field-effect transistor
Gr	Graphene
HIT	Heterojunction with intrinsic thin layer
HLDG	Hybrid Lorentz-Drude-Gaussian model
HNPs	Hexagonal-shaped nanoplates
HRTEM	High-Resolution Transmission Electron Microscopy
J-V	current density versus voltage
J_sc_	Short circuit current density
KPFM	Kelvin probe force microscopy
LSPR	Localized surface plasmon
NPs	Nanoparticles
NSs	Nanosheets
PCE	Power conversion efficiency
PDMS	Polydimethylsiloxane polymer
PL	Photoluminescence
PLD	Pulsed laser deposition
PPB	Particles per billions
PPM	Particles per millions
PSCs	Perovskite solar cells
R_a_	Resistance of the sensing element in the presence of atmospheric air
R_g_	Resistance of the sensing element in the presence of the target gas
RH	Relative humidity
SCs	Solar cells
SEM	Scanning electron microscopy
S_max_	The maximum value of the sensing response
SP	Surface potential
TCE	Transparent conducting electrode
TEM	Transmission electron microscopy
TD	Thermal decomposition
TMDs	Transition metal dichalcogenides
TRPL	Time-resolved photoluminescence
TVD	Thermal vapor deposition
TVS	Thermal vapor sulfurization
UV	Ultraviolet
V_oc_	Open circuit voltage
XPS	X-ray photoelectron spectroscopy
